# Metabolomics Study of the Biochemical Changes in the Plasma of Myocardial Infarction Patients

**DOI:** 10.3389/fphys.2018.01017

**Published:** 2018-08-29

**Authors:** Mingdan Zhu, Yanqi Han, Yu Zhang, Shaoqiang Zhang, Congcong Wei, Zidong Cong, Wuxun Du

**Affiliations:** ^1^Second Affiliated Hospital of Tianjin University of Traditional Chinese Medicine, Tianjin, China; ^2^Tianjin Engineering Laboratory of Quality Control Techniques for Traditional Chinese Medicine, Tianjin Institute of Pharmaceutical Research, Co., Ltd., Tianjin, China; ^3^Tianjin University of Traditional Chinese Medicine, Tianjin, China

**Keywords:** myocardial infarction, metabolomics, potential biomarkers, UPLC/ESI–Q-TOF/MS, principal component analysis (PCA)

## Abstract

Myocardial infarction (MI) is a common and multifactorial disease that has the highest morbidity and mortality in the world. Although a number of physiological, pathological, and functional parameters have been investigated, only scarce information regarding the changes of small metabolites in the plasma has been reported, and this lack of information may cause poor MI diagnosis and treatment. In the present study, we aimed to investigate the metabolic profiles of plasma samples from MI patients to identify potential disease biomarkers and to study the pathology of MI. Metabolic profiles of the plasma of 30 MI patients and 30 controls were obtained using ultra-performance liquid chromatography/electrospray ionization quadruple time-of-flight mass spectrometry. The resulting data were processed using pattern recognition approaches, including principal component analysis and partial least squares-discriminant analysis, to identify the metabolites that differed between the groups. Significant differences in the plasma levels of the following 10 metabolites were observed in the MI patients compared with the controls: phosphatidylserine, C16-sphingosine, *N*-methyl arachidonic amide, *N*-(2-methoxyethyl) arachidonic amide, linoleamidoglycerophosphate choline, lyso-PC (C18:2), lyso-PC (C16:0), lyso-PC (C18:1), arachidonic acid, and linoleic acid. The changes in these 10 biomarkers indicated perturbations of energy metabolism, phospholipid metabolism, and fatty acid metabolism in the MI patients. These findings hold promise to advance the treatment, diagnosis, and prevention of MI.

## Introduction

The WHO has declared that cardiovascular disease is a modern epidemic, and it is one of the leading causes of morbidity and mortality all over the world. Although the mortality rate is decreasing due to recent improvements in medical technology, the prevalence is steadily increasing (The World Health Report, 1997; Law et al., 2002). MI is one of the most frequently occurring cardiovascular conditions in both developed and developing countries and can be a major catastrophic event that leads to sudden death or hemodynamic deterioration (Murray and Lopez, 1997). The risk factors for MI are caused by interactions between environmental and genetic factors that include hypercholesterolemia, diabetes mellitus, hypertension, obesity, and smoking (Teslovich et al., 2010). Although research regarding the physiological, pathological, and functional parameters and the treatment and prognosis of MI has been conducted over the last decade, little information about changes in the small metabolites in the plasma has been reported, and this lack of information may be detrimental to the diagnosis and treatment of MI (Li et al., 2012).

Metabolomics is an emerging and powerful discipline concerning comprehensive analyses of small molecules (<1 kDa) and provides powerful methods to discover biomarkers in biological systems (Wang et al., 2012). Metabolomics is based on dynamic changes in low molecular weight metabolites in organisms and has been applied in clinical research, human nutrition, plant physiology, microbiology metabolism and environmental toxicology studies (Li et al., 2008). The metabolites often mirror the end results of genomic and protein perturbations due to disease, and these results are closely associated with phenotypic changes. Various analytical methods involving multivariate data analysis, such as PCA and PLS-DA, have been applied in metabolomic-based drug metabolism studies (Zhang et al., 2012a). Moreover, some advanced instruments, such as UPLC/ESI–Q-TOF/MS, have become the widely applied techniques in metabolomics studies. The UPLC/ESI–Q-TOF/MS system is not only capable of providing accurate fragment mass, precursor ion, and neutral loss information but also exhibits high peak capacity, sensitivity, and resolution (Zhang et al., 2012b). Metabolomics combined with advanced instruments and appropriate analytical methods provides support that enables us to explain the metabolites associated with MI.

In recent years, the studies on metabolic disorders of urine, serum, and plasma from MI-treated rats have been reported with the aims to interpret the biochemical process and evaluate the pharmacological actions of diverse drugs. Many potential MI biomarkers in urine, serum, and plasma samples of rats have been identified relating to inflammation, oxidative injury, energy metabolism, and hypertrophy which were considered as the most relevant pathological changes in the formation of MI (Liu Y.T. et al., 2013; Jiang et al., 2014). However, the biomarkers of plasma samples from MI patients with qi deficiency and blood stasis have still few been studied using a metabonomic approach. By comparing with the experiments of rats samples, the clinical samples can offer a more direct and convincing result on MI metabolic information.

In the present study, we investigated the metabolic profiles of plasma samples from MI patients to identify potential disease biomarkers and to research the pathology of MI. The plasma metabolic profiles of 30 MI patients and 30 controls were obtained using UPLC/ESI–Q-TOF/MS. The resulting data were processed with pattern recognition approaches that included PCA and PLS-DA to discover the differentially expressed metabolites.

## Materials and Methods

### Ethics Statement

Written informed consent was obtained from the patients and healthy people in the study. The experimental protocol was reviewed and approved by the Institutional Review Board of Second Affiliated Hospital of Tianjin University of TCM. Ethics review was approved by Ethics Committee of Xi Yuan Hospital of China Academy of Chinese Medical Sciences.

### Chemicals

HPLC-grade acetonitrile and methanol were purchased from Fisher (Pittsburgh, PA, United States). Formic acid (HPLC-grade) and LEA (HPLC-grade) were purchased from Sigma-Aldrich (St. Louis, MO, United States). Ultrapure water (18.2 MΩ) was prepared from distilled water using a Milli-Q water purification system (Millipore Laboratories, Bedford, MA, United States).

### Subjects

Thirty MI patients were recruited from the Second Affiliated Hospital of Tianjin University of TCM, China in the period from August 13, 2014 to August 12, 2015. Briefly, the selection criteria were as follows: (1) patients aged between 40 and 70 years; (2) patients with acute MI within the previous 1–6 months; (3) patients exhibiting qi deficiency and blood stasis; (4) patients who were conscious so that the clinical data could be collected; and (5) patients who provided written informed consent. The exclusion criteria for all patients were severe hypertension, hyperlipidemia, pulmonary dysfunction, arrhythmia, hepatosis, renal inadequacy, mental diseases, pregnant and breast-feeding women, and other diseases that would have affected the clinical observations. The subjects were confirmed according to the “Clinical Guideline of New Drugs of Traditional Chinese Medicine for the Treatment of Coronary Heart Disease.” The control group consisted of blood samples from 30 individuals who attended the hospital for routine physical check-ups, and the control subjects were age- and gender-matched to the MI patients.

### Plasma Sample Preparation

The blood samples were anticoagulated with natrium citricum and centrifuged at 13,000 rpm for 10 min to obtain plasma samples. The plasma samples (300 μL) were mixed with methanol (1200 μL) *via* vortexing for 2 min and then centrifuged at 4°C for 15 min at 13,000 rpm. The supernatants (1200 μL) were transferred to new 1.5-mL polypropylene tubes and evaporated to dryness using a vacuum drying oven. Next, 150 μL of 50% methanol was added to dissolve the residues in each tube. The solutions were filtered through 0.22-μm Millipore filters prior to sample injection.

### UPLC and MS Analyses

The chromatographic separation was performed on a Waters Acquity UPLC BEH C18 column (2.1 mm × 100 mm, 1.7 μm, Waters, Corp., Milford, MA, United States) using a Waters ACQUITY UPLC system equipped with a binary solvent delivery system and an autosampler. The column temperature was maintained at 30°C. UV detection was performed over the range of 190–400 nm. The mobile phase was composed of phase A (acetonitrile) and phase B (water with 0.1% formic acid). The gradient for the plasma samples was as follows: 0–4 min, 2–30% A; 4–5 min, 30–40% A; 5–8 min, 40–40% A; 8–14 min, 40–50% A; 14–18 min, 50–55% A; 18–22 min, 55–90% A; and 22–24 min of washing in 90% A. The proportion of phase A returned to 2% in 2 min, and the column was allowed to re-equilibrate for 5 min before the next injection. The flow rate was 0.4 mL/min, and 5 μL were injected into the column. The column eluent was directed to the mass spectrometer without splitting.

The mass spectrometry was performed with a Waters Q/TOF Premier Mass Spectrometer (Waters, Corp., Manchester, United Kingdom) coupled to an electrospray ionization source (ESI). The mass spectra were acquired in both the negative and positive ion voltage modes with the following parameters: capillary voltages, 2.5 kV (negative mode) and 3.0 kV (positive mode); sample cone voltage, 30 V; extraction cone voltage, 4.0 V; collision energy, 5 eV; desolvation gas (nitrogen was used as the drying gas) flow rate, 600 L/h; desolvation temperature, 300°C; cone gas rate, 50 L/h; source temperature, 100°C; and scan range, *m/z* 50–1000 Da. The scan time and inter-scan delay were set to 0.15 and 0.02 s, respectively. All data were acquired using a LockSpray interface (LEA, *m/z* 555.2931 for the positive mode and 553.2775 for the negative mode) to ensure accuracy and reproducibility at a concentration of 200 ng/mL and a flow rate of 20 μL/min.

### Multivariate Data Analysis and Data Processing

The original chromatographic peak data obtained from the UPLC/ESI–Q-TOF/MS system were recognized and matched with the MarkerLynx Application Manager (Waters, United States). The main parameters were as follows: retention time range, 0–24 min; mass range, 50–1000 Da; mass tolerance, 0.2 Da; minimum intensity, 1%; mass window, 0.05, retention time tolerance, 0.02 min; and noise elimination level, 6. Pattern recognition analyses are practical methods in metabolomic investigations, and the analyses used here included unsupervised PCA and supervised PLS-DA. The PLS-DA model was processed with Simca-P software (version 11.5, Demo, Umetrics, Umea, Sweden) as used to concentrate the group discrimination into the first component, while the remaining unrelated variation was contained in the subsequent components (Boccard and Rutledge, 2013). According to the significance values and screening of the score plots and loading plots, the potential biomarkers were identified.

### Biomarker Identification

The selected potential biomarkers were precisely identified, and the elemental compositions were generated with MarkerLynx based on the exact masses of the metabolites with high contribution scores. The MS/MS fragments of the biomarkers were obtained in a consistent manner. The following databases were used to identify the potential markers: ChemSpider^1^, MetFrag^2^, MassBank^3^, and PubChem^4^.

### Metabolic Pathway Analysis

The pathway analyses of the potential biomarkers were performed with database sources that included the Human Metabolome Database (HMDB^5^), the Kyoto Encyclopedia of Genes and Genomes (KEGG^6^), METLIN^7^, the Small Molecule Pathway Database (SMPD^8^), the LIPID Metabolites and Pathways Strategy (Lipid MAPS^9^), and the Scripps Center for Metabolomics and Mass Spectrometry.^10^

### Statistical Analysis

The statistical analyses were performed using SPSS software (version 16.0, Chicago, IL, United States), and statistical significance was set at *p* < 0.05. The multivariate statistical analyses were performed with the SIMCA-P 11.5 software package (Demo, Umetrics, Umeå, Sweden). The PLS-DA model was subsequently validated using cross-model validation and permutation.

## Results

### Clinical Characteristics of Subjects

Thirty MI patients and 30 controls were included in this study. The clinical characteristics of the MI patients and controls are listed in **Table 1**. The enrolled patients and controls were well-matched in terms of age, gender, and ethnicity, which indicated that the intra-group differences were mainly due to MI-related pathological variations. The biochemistry data revealed that the levels of the indicators TC and TG were higher in the MI patients than in the controls, and no significant differences were observed in the CK, CK-MB, or CRE levels.

**Table 1:** The basic characteristics of the subjects.

	Control	MI patients	*p*-value^a^
No. of subjects	30	30	–
Age, mean ± SD, years	55.23 ± 6.21	56.30 ± 6.70	0.525
Gender, male/female, no.	15/15	15/15	1.000
CK (U/L)	93.73 ± 44.24	97.53 ± 43.82	0.739
CK-MB (U/L)	14.43 ± 4.23	13.77 ± 3.33	0.500
CER (μmol/L)	74.41 ± 20.64	76.05 ± 19.67	0.754
TC (mmol/L)	3.79 ± 0.96	4.70 ± 1.18	0.002
TG (mmol/L)	1.11 ± 0.38	2.32 ± 0.59	0.000

*^*a*^Unpaired *t*-tests and χ^*2*^ tests were used to assess the difference between control and patients.*

### Validation of the UPLC-MS Method

The precision of the instrument and accuracy of method reflected the stability of the analysis, which were very important for guaranteeing the reliability of the acquired metabolomic data. In the present study, the injection precision was determined by repeatedly analyzing sets of six injections from the same samples. The RSDs of the peak areas were between 2.2 and 3.8%. To evaluate the influence of sample preparation on the stability of the data, six parallel samples were prepared using the same preparation protocol. The retention times of the peaks remained almost unchanged, and the RSDs of the peak intensities of the major signals were below 3.9%. These results indicate that the repeatability of sample preparation met the requirements for metabolomics analysis.

### Metabolic Profiling Analysis

**FIGURE 1:**
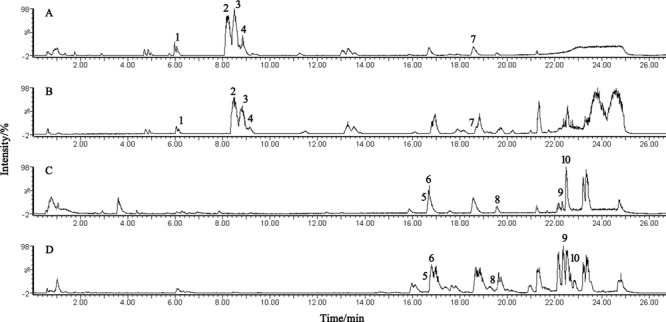
UPLC-Q-TOF/MS analysis of the plasma. **(A,B)** BPI chromatograms of plasma from the control and MI patients acquired in positive mode. **(C,D)** BPI chromatograms of plasma from the control and MI patients acquired in negative mode.

**FIGURE 2:**
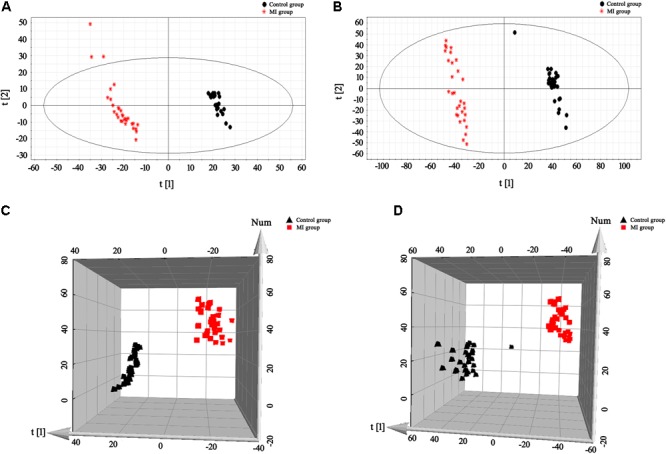
PLS-DA score plots for the plasma samples of the control and MI patients. **(A,B)** 2-D plots of the plasma results in positive and negative modes, respectively. **(C,D)** 3-D plots of the plasma results in positive and negative modes, respectively. Classification shows a clear distinction between the control group (black) and MI group (red).

**FIGURE 3:**
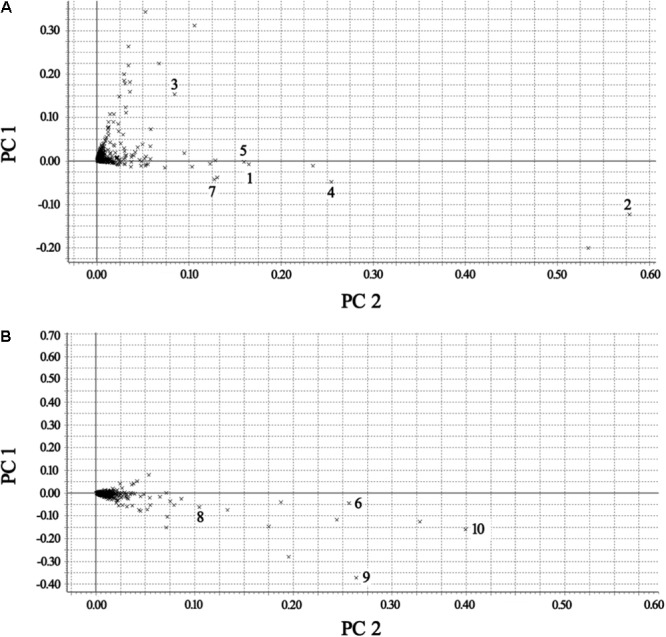
Loading plots of the metabolomes of the plasma from the control and MI patients. **(A)** Positive mode. **(B)** Negative mode [1 – phosphatidylserine; 2 – C16-sphingosine; 3 – *N*-methyl arachidonic amide; 4 – *N*-(2-methoxyethyl) arachidonic amide; 5 – linoleamidoglycerophosphate choline; 6 – lyso-PC (C18:2); 7 – lyso-PC (C16:0); 8 – lyso-PC (C18:1); 9 – arachidonic acid; and 10 – linoleic acid].

The BPI current chromatograms from the MI patients and controls in both the negative and positive ESI modes are shown in **Figure 1**. All of the retention time, peak intensity, and exact mass data were imported into the MassLynx software, and the data from MassLynx were directly imported into the Simca-P software for multiple statistical analyses. As shown in **Figure 2**, the analysis of the PLS-DA score plots of the first and second principal components (PC1 and PC2, respectively) in the positive and negative modes truly reflected the differences between the MI patients and controls and revealed that the metabolic profiles of the control and patient groups were clearly separated. All of the samples were classified into two main groups, and none were misclassified, which demonstrates that the constituents of the samples from the different stages were significantly different. The *R2X, R2Y*, and *Q2* of this PLS-DA model were 0.210, 0.994, 0.994 and 0.173, 0.990, 0.968 in positive and negative modes, respectively. Loading plots were utilized to reveal the contributions of each principal component. The distance from the origin of the loading plots to the marker is indicative of the significance of the marker, and higher values indicate more significant markers. As shown in **Figure 3**, 10 compounds strongly contributed to the clusters in both the positive and negative modes (variables with statistical significant difference *p* < 0.05) and were identified as responsible for the separation between the MI patients and control groups. Therefore, 10 components were regarded as potential biomarkers.

### Identification of Potential Biomarkers

The presumed molecular formulas were searched in HMDB and other databases to identify the possible chemical constitutions. Furthermore, the MS/MS data were screened to determine the potential structures of the ions. To illustrate the identification of the metabolites, we selected the ion at *t*_R_ = 18.59 min (*m/z* 496.3356) as an example that will be described. The [M + H] 496.3356 demonstrated an odd number of nitrogen atoms, and its molecular formula was speculated to be C_24_H_50_NO_7_P based on analyses of the elemental composition and fractional isotope abundance. In the positive mode, the MS/MS figure contained fragmentations of [M + H-H_2_O]^+^ (*m/z* 478.3), [M + H-C_5_H_13_NO_4_P]^+^ (*m/z* 313.2), and [M + H-C_19_H_37_NO_2_]^+^ (*m/z* 184.0). Finally, the metabolite was tentatively identified as LPC (16:0) based on the database searches. The data in the literature were also used to confirm this result (Liu P. et al., 2013).

According to the protocol detailed above, 10 endogenous metabolites in the plasma were tentatively identified and are summarized in **Table 2**. The metabolites of *N*-methyl arachidonic amide (No. 3) and AA (No. 9) were obviously up-regulated (*p* < 0.05) in the MI group compared with the control group, and the levels of phosphatidylserine (No. 1), C16-sphingosine (No. 2), *N*-(2-methoxyethyl) arachidonic amide (No. 4), linoleamidoglycerophosphate choline (No. 5), lyso-PC (C18:2) (No. 6), lyso-PC (C16:0) (No. 7), lyso-PC (C18:1) (No. 8), and linoleic acid (No. 10) were significantly decreased (*p* < 0.05) in the MI group.

**Table 2:** MS/MS data in the positive and negative ESI modes and the identification results for the biomarkers.

Peak no.	RT (min)	Mode	*m/z*	Formula	Identification	Significance	Content variance	Percentage change	Reported	Metabolic pathway
1	6.07	[M + H]^+^	568.3423	C_26_H_50_NO_10_P	Phosphatidylserine	0.2332	↓	33.73	Yes	Glycerophospholipid metabolism
2	8.19	[M + H]^+^	274.2722	C_16_H_35_NO_2_	C16-Sphingosine	0.4179	↓	12.85	Yes	Sphingolipid metabolism
3	8.70	[M + H]^+^	318.2017	C_21_H_35_NO	*N*-methyl arachidonic amide	0.1240	↑	144.8	No	Arachidonic acid metabolism
4	8.80	[M + H]^+^	362.3266	C_23_H_39_NO_2_	*N*-(2-methoxyethyl) arachidonic amide	0.1830	↓	52.14	No	Arachidonic acid metabolism
5	16.72	[M + H]^+^	520.3378	C_26_H_50_NO_7_P	Linoleamidoglycerophosphate choline	0.1129	↓	17.73	No	Glycerophospholipid metabolism
6	16.76	[M + HCOO]^-^	564.3354	C_27_H_51_NO_9_P	Lyso-PC (C18:2)	0.1836	↓	42.28	Yes	Glycerophospholipid metabolism
7	18.59	[M + H]^+^	496.3356	C_24_H_50_NO_7_P	Lyso-PC (C16:0)	0.0947	↓	32.22	Yes	Glycerophospholipid metabolism
8	19.61	[M + HCOO]^-^	566.3452	C_27_H_53_NO_9_P	Lyso-PC (C18:1)	0.0854	↓	85.12	Yes	Glycerophospholipid metabolism
9	22.37	[M-H]^-^	303.2318	C_20_H_32_O_2_	Arachidonic acid	0.3225	↑	299.42	Yes	Arachidonic acid metabolism
10	22.52	[M-H]^-^	279.2293	C_18_H_32_O_2_	Linoleic acid	0.2874	↓	70.48	Yes	Fatty acid β-oxidation metabolism

### Metabolic Pathway Analysis

Pattern recognition analysis of the metabolites revealed that an obvious separation of the MI model and the control group was achieved. Metabolite profiling focuses on the analysis of a group of metabolites that are related to specific metabolic pathways that are involved in biological states. Based on the identified potential biomarkers from LC–MS, the perturbed metabolic pathways in the MI patients were identified based on the KEGG pathway database. The metabolites were primarily involved in alterations in glycerophospholipid metabolism (Nos. 1, 5, 6, 7, and 8), AA metabolism (Nos. 3, 4, and 9), sphingolipid metabolism (No. 2), and fatty acid β-oxidation metabolism (No. 10).

## Discussion

In this study, UPLC/ESI–Q-TOF/MS-based metabolomics study was used to investigate the metabolic profiles of plasma samples from MI patients to identify potential disease biomarkers and to study the pathology of MI. First, the clinical and biochemistry characteristics of MI patients and controls were compared, which indicates the difference in TC and TG between two groups was significant. Moreover, 10 compounds, namely, phosphatidylserine, C16-Sphingosine, *N*-methyl arachidonic amide, *N*-(2-methoxyethyl) arachidonic amide, linoleamidoglycerophosphate choline, Lyso-PC (C18:2), Lyso-PC (C16:0), Lyso-PC (C18:1), AA, and linoleic acid, were identified and determined to be potential biomarkers in the MI patients. Furthermore, Metabolic pathway analysis of these metabolites suggested that glycerophospholipid metabolism, AA metabolism, sphingolipid metabolism, and fatty acid β-oxidation metabolism were the most significantly metabolic pathways in the MI patients.

Metabolomics-based biomaker study offers a novel and sensitive technique in the biomarker discovery for early diagnosis and treatment (Dang et al., 2018). Comprehensive strategies attract increasing attentions in MI study (Shah et al., 2012a; Forssen et al., 2017). GC-MS-based tissue metabolomics study of MI rats suggested that 22 metabolites were identified to be different between the infarcted myocardia and non-infarcted myocardia (Wang et al., 2017). A non-targeted UHPLC-Q-TOF/MS metabolomics approach was applied to confirm the alterations in CHD patients and metabolites, namely, 4-pyridoxic acid, PG (20:3/2:0) and lithocholic acid were identified, which exhibited strong correlations with CHD (Li et al., 2017). In the present study, we combined UPLC/ESI–Q-TOF/MS and metabolomics method to investigate the biomarker of the MI patients. Based on the results, we speculated that the differences (shown in **Table 2**) in the plasma might denote potential biomarker targets for differentiating MI pathological and control states. Among the determined 10 metabolites, phosphatidylserine, C16-sphingosine, Lyso-PC (C18:2), Lyso-PC (C16:0), Lyso-PC (C18:1), AA, and linoleic acid have been reported to be the biomarkers for cardiovascular disease (Liu Y.T. et al., 2013; Jiang et al., 2014; Park et al., 2015), and we determined *N*-methyl arachidonic amide, *N*-(2-methoxyethyl) arachidonic amide, and linoleamidoglycerophosphate choline to be the new biomarkers for MI.

The levels of phosphatidylserine, linoleamidoglycerophosphate choline, Lyso-PC (C18:2), Lyso-PC (C16:0), and Lyso-PC (C18:1), which were involved in the glycerophospholipid metabolism, were downregulated in MI patients compared with control subjects in our study. Glycerophospholipids are precursors of lipid mediators that are involved in the signal transduction process. Lyso-PC (C18:2) (No. 6), lyso-PC (C16:0) (No. 7), and lyso-PC (C18:1) (No. 8) are glycerophospholipids, which are important components of all cell membranes (Fuly et al., 2007). Phospholipase A2 can be activated during the breakdown of the membrane, which results in further decreases in the generation of lyso-PCs (Glukhova et al., 2015). The degradation of glycerophospholipids by phospholipase A2 generates LPC and AA. LPC is then enzymatically converted to LPA (Wymann and Schneiter, 2008) and has been identified as risk factor biomarkers for coronary artery disease (Zhang et al., 2017). Moreover, increased AA (No. 9) in adipose tissue is associated with an increased risk of non-fatal acute MI (Wang et al., 2014). Specifically, AA is oxygenated by cyclooxygenase form prostaglandins or by lipoxygenase enzymes to for leukotrienes, which can mediate or modulate inflammatory reactions (Jabbour et al., 2009).

Sphingolipids (phytosphingosine and sphingosine, No. 2) are components of cellular membranes in eukaryotic cells (Lynch, 2012). Ceramide and S1P are generated from phytosphingosine. Ceramide is converted into sphingosine and sphingomyelins (Gault et al., 2010). The decrease in sphingosine observed in this study might reflect either a reduction in the synthesis of the metabolite or its rapid consumption for the increased synthesis of S1P or sphingomyelins. Studies have found that S1P plays an important role in vascular maturation, and S1P has been implicated in the pathophysiology of atherosclerosis and wound healing (Watterson et al., 2003).

Fatty acids are the largest energy reserve in the body and supply energy-yielding substrates *via* β-oxidation in the mitochondria and peroxisomes. Fatty acids have also been regarded as independent predictors of cardiovascular events (Shah et al., 2012b). The reduction of linoleic acid (No. 10) observed in this study indicates that the β-oxidation of the unsaturated fatty acid was inhibited, and such inhibition contributes to myocardial damage (Hjelte and Nilsson, 2005).

In conclusion, our metabolomics-based biomaker study results showed that 10 identified metabolites, related to energy metabolism, phospholipid metabolism, and fatty acid metabolism, played significantly role in MI patients. Interestingly, we observed glycerophospholipid metabolism emerged as the most significantly disturbed pathway, which was consistent with previous findings (Lu et al., 2017). Consequently, the identified metabolites, especially phosphatidylserine, linoleamidoglycerophosphate choline, Lyso-PC (C18:2), Lyso-PC (C16:0), and Lyso-PC (C18:1), may be better metabolites to forecast the risk for MI patients. Moreover, comprehensive metabolomics study may offered a technique in biomarker discovery and understanding disease mechanisms.

## Conclusion

Metabolomics provides useful tools for identifying differences in metabolic pathways between patients and controls and predicting and discovering biomarkers for the prediction of diseases. In the present UPLC/ESI–Q-TOF/MS-based metabolomics study, we obtained more detailed information about the metabolic changes that occur in patients. In this study, we investigated the metabolic profiles of plasma samples from 30 MI patients and 30 controls using UPLC/ESI–Q-TOF/MS spectroscopy coupled with multivariate statistical analyses that included PCA and PLS-DA. The results revealed that endogenous metabolites are altered in MI patients. The 10 identified metabolites that are potentially associated with perturbations of energy metabolism, phospholipid metabolism, and fatty acid metabolism will contribute to progress related to MI. These findings hold promise to advance the treatment, diagnosis, and prevention of MI.

## Author Contributions

MZ and WD contributed conception and design of the study. SZ, CW, and ZC performed the statistical analysis. MZ and YH wrote the first draft of the manuscript. All authors contributed to manuscript revision, read, and approved the submitted version.

## Conflict of Interest Statement

YH was employed by company Tianjin Institute of Pharmaceutical Research, Co., Ltd. The remaining authors declare that the research was conducted in the absence of any commercial or financial relationships that could be construed as a potential conflict of interest.

## Footnotes

1 http://www.chemspider.com

2 http://msbi.ipb-halle.de/MetFrag/

3 http://www.massbank.jp

4 http://ncbi.nlm.nih.gov/

5 http://www.hmdb.ca/

6 https://www.genome.jp/kegg/

7 http://metlin.scripps.edu/

8 http://www.smpdb.ca/

9 http://www.lipidmaps.org/

10 http://masspec.scripps.edu/index.php

## Abbreviations

AA: arachidonic acid
BPI: basic peak ion
CK: creatine kinase
CK-MB: creatine kinase isoenzyme
CRE: creatinine
LEA: leucine enkephalinamide acetate
LPA: lysophosphatidic acid
LPC: lysophosphatidylcholine
MI: myocardial infarction
PCA: principal component analysis
PLS-DA: partial least squares-discriminant analysis
RSDs: relative standard deviations
S1P: sphingosine 1 phosphate
TC: total cholesterol
TCM: traditional Chinese medicine
TG: triglyceride
UPLC/ESI–Q-TOF/MS: ultra-performance liquid chromatography/electrospray ionization quadruple time-of-flight mass spectrometry
WHO: World Health Organization

## References

[B1] Boccard J. Rutledge D. N. (2013). A consensus orthogonal partial least squares discriminant analysis (OPLS-DA) strategy for multiblock Omics data fusion. *Anal. Chim. Acta* 769 30–39. 10.1016/j.aca.2013.01.022 23498118

[B2] Dang V. T. Huang A. Werstuck G. H. (2018). Untargeted metabolomics in the discovery of novel biomarkers and therapeutic targets for atherosclerotic cardiovascular diseases. *Cardiovasc. Hematol. Disord. Drug Targets* 10.2174/1871529X18666180420170108 [Epub ahead of print]. 29683098

[B3] Forssen H. Patel R. Fitzpatrick N. (2017). Evaluation of machine learning methods to predict coronary artery disease using metabolomics data. *Stud. Health Technol. Inform.* 235 111–115. 28423765

[B4] Fuly A. L. Machado A. L. Castro P. (2007). Lysophosphatidylcholine produced by the phospholipase A2 isolated from *Lachesis muta* snake venom modulates natural killer activity as a protein kinase C effector. *Toxicon* 50 400–410. 10.1016/j.toxicon.2007.04.008 17537472

[B5] Gault C. R. Obeid L. M. Hannun Y. A. (2010). An overview of sphingolipid metabolism: from synthesis to breakdown. *Adv. Exp. Med. Biol.* 688 1–23. 10.1007/978-1-4419-6741-1_1 20919643 PMC3069696

[B6] Glukhova A. Hinkovska-Galcheva V. Kelly R. (2015). Structure and function of lysosomal phospholipase A2 and lecithin:cholesterol acyltransferase. *Nat. Commun.* 6:6250. 10.1038/ncomms7250 25727495 PMC4397983

[B7] Hjelte L. E. Nilsson A. (2005). Arachidonic acid and ischemic heart disease. *J. Nutr.* 135 2271–2273. 10.1093/jn/135.9.2271 16140910

[B8] Jabbour H. N. Sales K. J. Catalano R. D. Norman J. E. (2009). Inflammatory pathways in female reproductive health and disease. *Reproduction* 138 903–919. 10.1530/REP-09-0247 19793840

[B9] Jiang M. Kang L. Wang Y. (2014). A metabonomic study of cardioprotection of ginsenosides, schizandrin, and ophiopogonin D against acute myocardial infarction in rats. *BMC Complement. Altern. Med.* 14:350. 10.1186/1472-6882-14-350 25249156 PMC4182767

[B10] Law M. R. Watt H. C. Wald N. J. (2002). The underlying risk of death after myocardial infarction in the absence of treatment. *Arch. Intern. Med.* 162 2405–2410. 10.1001/archinte.162.21.2405 12437397

[B11] Li C. Pei F. Zhu X. Duan D. D. Zeng C. (2012). Circulating microRNAs as novel and sensitive biomarkers of acute myocardial infarction. *Clin. Biochem.* 45 727–732. 10.1016/j.clinbiochem.2012.04.013 22713968 PMC3965350

[B12] Li H. Jiang Y. He F. C. (2008). Recent development of metabonomics and its applications in clinical research. *Yi Chuan* 30 389–399. 10.3724/SP.J.1005.2008.00389 18424407

[B13] Li Y. Zhang D. He Y. (2017). Investigation of novel metabolites potentially involved in the pathogenesis of coronary heartdisease using a UHPLC-QTOF/MS-based metabolomics approach. *Sci. Rep.* 7:15357. 10.1038/s41598-017-15737-3 29127404 PMC5681629

[B14] Liu P. Duan J. Wang P. (2013). Biomarkers of primary dysmenorrhea and herbal formula intervention: an exploratory metabonomics study of blood plasma and urine. *Mol. Biosyst.* 9 77–87. 10.1039/c2mb25238d 23111557

[B15] Liu Y. T. Jia H. M. Chang X. (2013). The metabolic disturbances of isoproterenol induced myocardial infarction in rats based on a tissue targeted metabonomics. *Mol. Biosyst.* 9 2823–2834. 10.1039/c3mb70222g 24057015

[B16] Lu J. Chen B. Chen T. (2017). Comprehensive metabolomics identified lipid peroxidation as a prominent feature in humanplasma of patients with coronary heart diseases. *Redox Biol.* 12 899–907. 10.1016/j.redox.2017.04.032 28472752 PMC5415551

[B17] Lynch D. V. (2012). Evidence that sphingolipid signaling is involved in responding to low temperature. *New Phytol.* 194 7–9. 10.1111/j.1469-8137.2012.04078.x 22364118

[B18] Murray C. J. Lopez A. D. (1997). Alternative projections of mortality and disability by cause 1990-2020: global burden of disease study. *Lancet* 349 1498–1504. 10.1016/S0140-6736(96)07492-2 9167458

[B19] Park J. Y. Lee S. H. Shin M. J. Hwang G. S. (2015). Alteration in metabolic signature and lipid metabolism in patients with angina pectoris and myocardial infarction. *PLoS One* 10:e0135228. 10.1371/journal.pone.0135228 26258408 PMC4530944

[B20] Shah S. H. Kraus W. E. Newgard C. B. (2012a). Metabolomic profiling for the identification of novel biomarkers and mechanisms related to common cardiovascular diseases: form and function. *Circulation* 126 1110–1120. 10.1161/CIRCULATIONAHA.111.060368 22927473 PMC4374548

[B21] Shah S. H. Sun J. L. Stevens R. D. (2012b). Baseline metabolomic profiles predict cardiovascular events in patients at risk for coronary artery disease. *Am. Heart J.* 163 844.e1–850.e1. 10.1016/j.ahj.2012.02.005 22607863

[B22] Teslovich T. M. Musunuru K. Smith A. V. Edmondson A. C. Stylianou I. M. Koseki M. (2010). Biological, clinical and population relevance of 95 loci for blood lipids. *Nature* 466 707–713. 10.1038/nature09270 20686565 PMC3039276

[B23] The World Health Report. (1997). 1997–conquering suffering, enriching humanity. *World Health Forum* 18 248–260. 9478137

[B24] Wang X. Wang D. Wu J. (2017). Metabolic characterization of myocardial infarction using GC-MS-based tissue metabolomics. *Int. Heart J.* 58 441–446. 10.1536/ihj.16-432 28484125

[B25] Wang X. Yang B. Sun H. Zhang A. (2012). Pattern recognition approaches and computational systems tools for ultra performance liquid chromatography-mass spectrometry-based comprehensive metabolomic profiling and pathways analysis of biological data sets. *Anal. Chem.* 84 428–439. 10.1021/ac202828r 22132738

[B26] Wang Y. Li C. Liu Z. (2014). DanQi Pill protects against heart failure through the arachidonic acid metabolism pathway by attenuating different cyclooxygenases and leukotrienes B4. *BMC Complement. Altern. Med.* 14:67. 10.1186/1472-6882-14-67 24555740 PMC3933388

[B27] Watterson K. Sankala H. Milstien S. Spiegel S. (2003). Pleiotropic actions of sphingosine-1-phosphate. *Prog. Lipid Res.* 42 344–357. 10.1016/S0163-7827(03)00015-8 12790117

[B28] Wymann M. P. Schneiter R. (2008). Lipid signalling in disease. *Nat. Rev. Mol. Cell Biol.* 9 162–176. 10.1038/nrm2335 18216772

[B29] Zhang A. Sun H. Han Y. Yuan Y. Wang P. Song G. (2012a). Exploratory urinary metabolic biomarkers and pathways using UPLC-Q-TOF-HDMS coupled with pattern recognition approach. *Analyst* 137 4200–4208. 10.1039/c2an35780a 22852134

[B30] Zhang A. Sun H. Wang P. Han Y. Wang X. (2012b). Modern analytical techniques in metabolomics analysis. *Analyst* 137 293–300. 10.1039/C1AN15605E 22102985

[B31] Zhang X. Z. Zheng S. X. Hou Y. M. (2017). A non-targeted liquid chromatographic-mass spectrometric metabolomics approach for association with coronary artery disease: an identification of biomarkers for depiction of underlying biological mechanisms. *Med. Sci. Monit.* 23 613–622. 10.12659/MSM.896298 28151921 PMC5301958

